# The Corrosion Inhibition Performance and Synergistic Adsorption Mechanism of a Novel Quinoline-Based Inhibitor for J55 Steel in HCl Solution

**DOI:** 10.3390/molecules31213881

**Published:** 2026-10-27

**Authors:** Sanbao Dong, Jinhua Li, Haiwen Hu, Jiakui Chen, Yanmei Hou, Weiwei Han

**Affiliations:** 1 College of Chemistry and Chemical Engineering, Xi’an Petroleum University, No. 18, East Dianzi 2nd Road, Xi’an 710065, China; lijinhua0107@126.com (J.L.); chenjiakui113@126.com (J.C.); houyanmei@zjdx16.wecom.work (Y.H.); hanweiwei@xsyu.edu.cn (W.H.); 2 Shaanxi University Engineering Research Center of Oil and Gas Field Chemistry, Xi’an Petroleum University, Xi’an 710065, China; 3 Institute of Carbon Neutrality Future Technology, Xi’an Petroleum University, Xi’an 710065, China; hhwdodo@126.com

**Keywords:** quaternary ammonium salt corrosion inhibitor, static weight loss, electrochemical measurement, molecular dynamics simulation

## Abstract

Novel and high-efficiency corrosion inhibitors are indispensable in acidizing stimulation, which is a key operation to increase oil production. In this study, a novel corrosion inhibitor, alkynyl bisquinolinium quaternary ammonium salt (ABQ) containing both a quinoline ring and alkynyl groups, was first developed through a quaternization reaction using quinoline and 1,4-dichlorobutyne. ABQ was then compounded with 3-phenyl-2-propyn-1-ol (PPA). ABQ-PPA’s corrosion inhibition performance and mechanism for J55 steel in a hydrochloric acid medium were systematically investigated through static weight-loss tests, electrochemical measurements, SEM characterization, thermodynamic adsorption analysis, and molecular dynamics (MD) simulations. The results indicated that the 0.5% ABQ-PPA mixture (0.45% ABQ and 0.05% PPA, mass ratio 4.5: 0.5) produced superior inhibition properties (up to 99.89%). Electrochemical measurements demonstrated that the ABQ-PPA complex could simultaneously suppress both anodic and cathodic reactions, acting as a mixed-type inhibitor with predominant anodic inhibition. Adsorption isotherm analysis revealed that ABQ-PPA’s adsorption onto the J55 steel surface followed the Langmuir monolayer adsorption model, with a mixed adsorption mode involving both physisorption and chemisorption. Molecular dynamics simulations further revealed that ABQ-PPA molecules formed a compact and orderly mixed adsorption layer on the Fe surface, displacing interfacial water molecules and blocking corrosion attack from corrosive media.

## 1. Introduction

Acidizing treatment, one of the core technologies for enhancing oil and gas reservoir productivity and injectivity, involves injecting certain acids into low-permeability formations to dissolve pore-plugging materials and improve near-wellbore permeability [1,2,3]. However, a long-standing challenge associated with this technology is the severe corrosion risk to metal equipment, downhole tools, and surface facilities caused by the acid. Therefore, novel and high-efficiency corrosion inhibitors for acid working fluids have become essential measures to ensure the safe and efficient implementation of acidizing treatments [4].

Among corrosion inhibitors, nitrogen-containing triazole compounds can accelerate low-temperature zinc phosphating of microalloyed steel, highlighting the key role of heteroatoms in surface pretreatment and corrosion protection [5]. Natural additives containing heteroatoms and π-electron systems can effectively inhibit the oxidation of austenitic stainless steel, and DFT calculations confirmed that heteroatoms and aromatic rings are the main adsorption sites [6]. These results indicate that heteroatoms (N, O, S, P) and π-conjugated systems are crucial for strong adsorption and corrosion inhibition and that the combination of DFT and experiments is an effective approach to elucidate substrate–inhibitor interactions [6]. Quaternary ammonium salt compounds have become one of the most widely applied acidizing corrosion inhibitor systems due to their cationic nature, which facilitates the electrostatic adsorption of inhibitor molecules onto negatively charged metal surfaces, along with their high structural tunability and favorable thermal stability [7]. Specifically, N-containing heterocyclic quaternary ammonium salts (e.g., quinoline-based ones), which are also positively charged, preferentially interact with and bond to metal surfaces due to the high electron-density cloud surrounding the substituents on the heterocyclic ring, thus significantly enhancing corrosion inhibition performance [8]. For example, to protect carbon steel against 1 M HCl, Daoud et al. [9] synthesized two novel heterocyclic quinoline-derived corrosion inhibitors, both of which produced inhibition efficiencies exceeding 95% in 1 M HCl at 25 °C, indicating favorable adsorption and protective performance on carbon steel in an HCl solution environment. Feng et al. [10] synthesized 13 types of 8-hydroxyquinoline derivatives and tested them with N80 carbon steel in a 1 M HCl solution at 60 °C, among which the highest inhibition efficiency reached 91.92%, indicating that they can form a protective film on the steel surface through strong adsorption, effectively resisting acidic corrosion. However, the testing temperature for these two quinoline-derived corrosion inhibitors needs to be further increased.

Besides quinoline-derived quaternary ammonium salt corrosion inhibitors—which depend on the electrostatic attraction between the molecules and the metal surface—another type of corrosion inhibitor containing alkyne groups has also been extensively studied and reported. These alkynol-type corrosion inhibitors spontaneously adsorb onto metal surfaces via the π electrons of the triple bond, followed by cathodic hydrogenation, dehydration of the hydroxyl group to form dienes, and subsequent polymerization and crosslinking to generate a protective film, thereby improving the compactness and durability of the film layer [11,12]. Mainier et al. [13] tested the corrosion inhibition performance of propargyl alcohol on AISI 317L stainless steel in 5% and 10% hydrochloric acid at 25–55 °C, achieving a maximum inhibition efficiency of 99.5% at a dosage of 1%, indicating that it can effectively inhibit acidic corrosion. Shatirova et al. [14] synthesized a series of propargyl-containing phosphoric and phosphonic acid derivatives, which achieved an inhibition efficiency of up to 99.5% for 08kp steel in 17% HCl at 60 °C at a dosage of 1%. However, to the best of our knowledge, there are no reported corrosion inhibitor molecules containing both quinoline rings and alkynyl groups. Based on the working mechanisms of the quinoline ring and alkynyl groups, it is reasonable to expect that a molecule containing both quinoline rings and alkynyl groups would exhibit better corrosion inhibition performance, which motivated us to investigate whether there is synergy between the quinoline ring and alkynyl groups in a corrosion inhibitor molecule.

Based on the above design strategy, a novel alkynyl bis-quinolinium quaternary ammonium salt compound (ABQ) was developed via a quaternization reaction using quinoline and 1,4-dichlorobutyne as raw materials. The quinoline rings, serving as electron donors, provide a favorable conjugated system, and the introduced alkynyl functional group is expected to enhance the chemical bonding trend between the molecule and the J55 steel surface. This paper focuses on the synthesis and corrosion inhibition performance evaluation of ABQ and ABQ-PPA for J55 steel in acidizing media. The effects of concentration, temperature, and other factors on the inhibition ability were also investigated. SEM characterization, adsorption isotherm analysis, and molecular dynamics (MD) simulations were performed to shed light on ABQ-PPA’s underlying mechanism.

## 2. Results

### 2.1. Synthesis of ABQ

**Figure 1:**
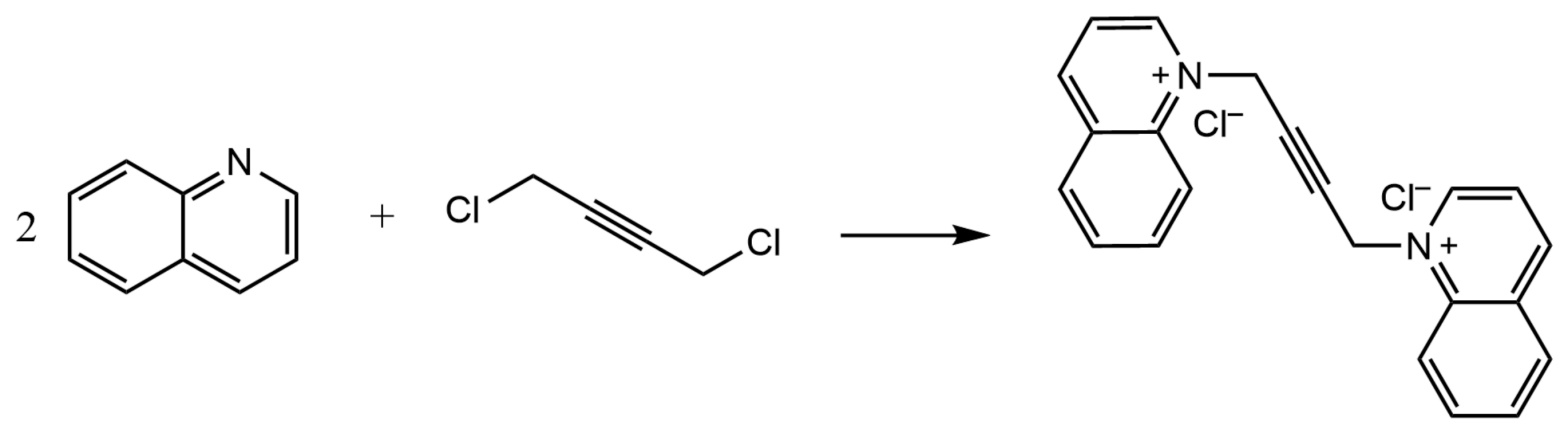
Synthetic route of ABQ.

Quinoline and 1,4-dichloro-2-butyne were accurately weighed at a molar ratio of 2.1:1 and dissolved in toluene in a round-bottom flask. The reaction was carried out at 120 °C under constant stirring for 12 h. Then, the resulting mixture was recrystallized to obtain the crude ABQ product. The crude product was further purified with a mixed solvent of ethanol and petroleum ether (volume ratio 1:9) to remove impurities. The residue in the flask after purification was transferred to a watch glass using a small amount of ethanol and then dried at 50 °C for 4 h to remove the residual ethanol. The product was finally placed in a desiccator and sealed for storage. Figure 1 displays the synthetic route of ABQ.

#### FT-IR Analysis of ABQ

**Figure 2:**
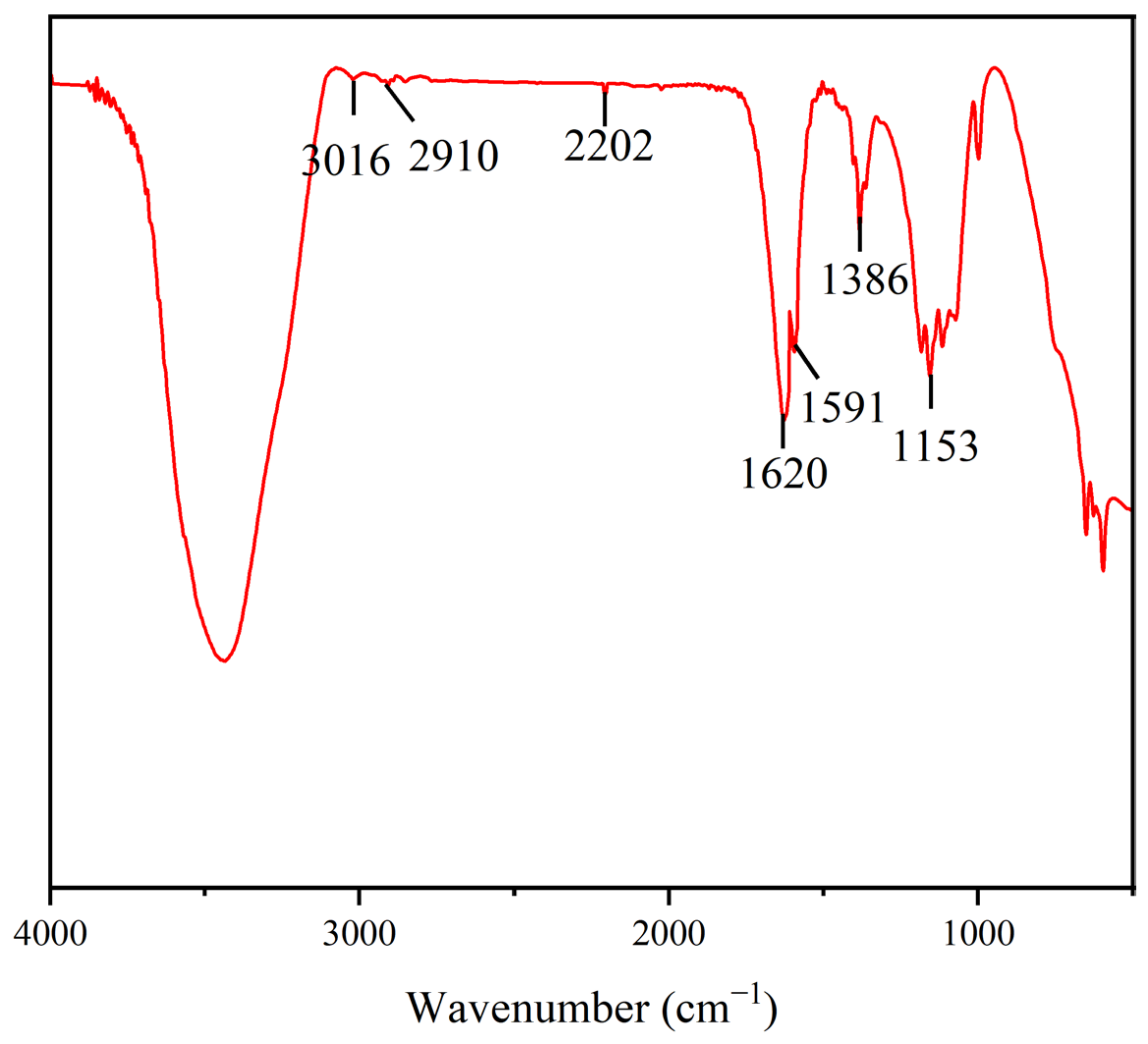
ABQ’s FT-IR spectrum.

The FT-IR spectrum of ABQ is shown in Figure 2. The absorption band at 2910 cm^−1^ corresponded to the C-H stretching vibration. The peak at 2202 cm^−1^ was attributed to the stretching vibration of the C≡C triple bond. The bands at 1620 cm^−1^ and 1591 cm^−1^ were from the C=C (from the aromatic ring) stretching vibration and the C=N stretching vibration, respectively. The absorption at 1386 cm^−1^ corresponded to the N^+^-CH_2_ bond’s bending vibration, while the 1153 cm^−1^ band was generated by the C-C skeletal vibration. These spectral features verified the successful preparation of ABQ.

### 2.2. Static Weight-Loss Tests

**Figure 3:**
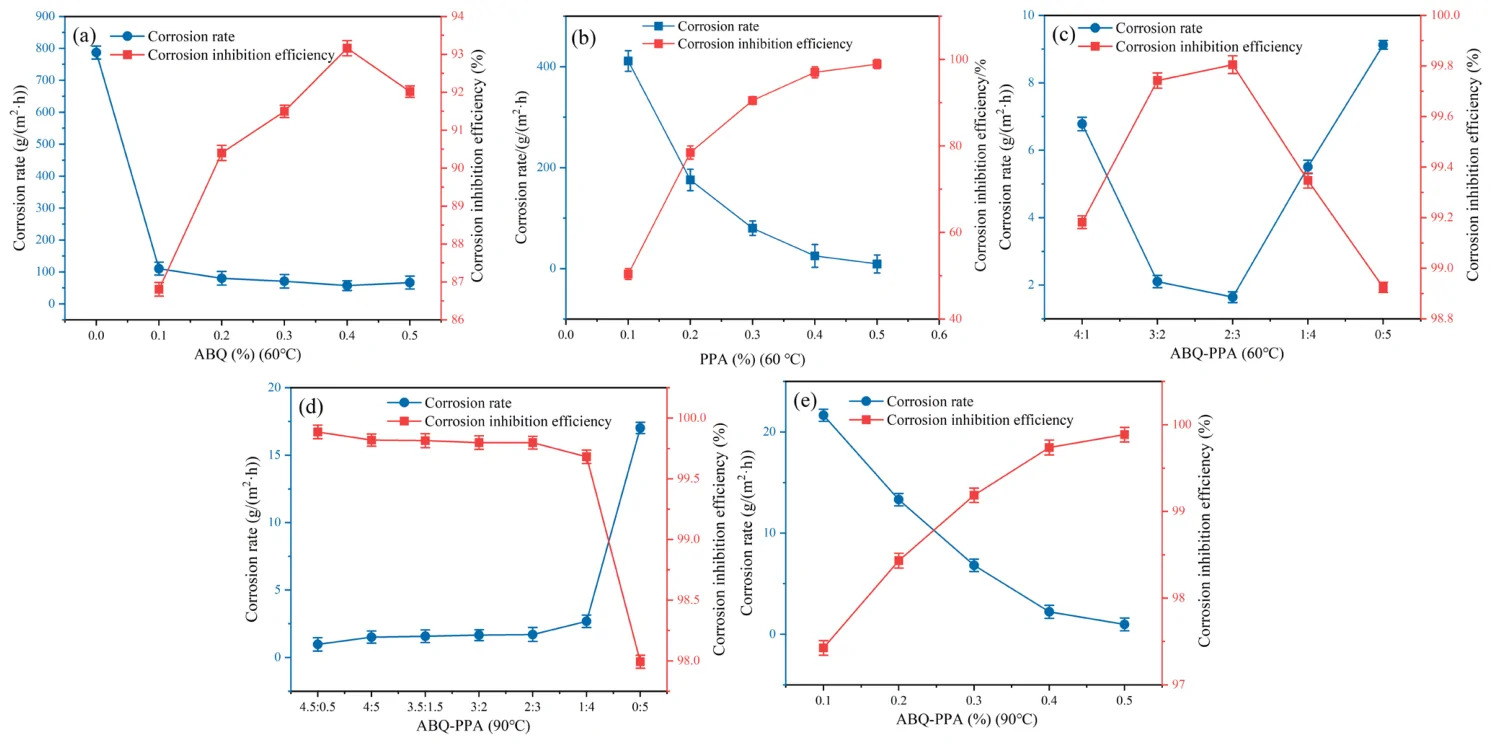
The protection performance of ABQ alone at 60 °C (**a**), PPA alone at 60 °C (**b**), ABQ-PPA at 60 °C (**c**), ABQ-PPA with different mass ratios at 90 °C (**d**), and ABQ-PPA with different total concentrations at 90 °C (**e**) for J55 steel in 20% HCl.

Figure 3 presents the evaluation results of the corrosion inhibition properties of different inhibition systems for J55 steel in a 20% HCl solution. As shown in Figure 3a, in a 20% HCl solution at 60 °C, ABQ’s inhibition efficiency alone reached up to 93.16%, with a corresponding corrosion rate of 56.98 g/(m^2^·h). Under strongly acidic (20% HCl) conditions, the quinoline nitrogen of ABQ is readily protonated to form a cation, which can adsorb onto the negatively charged steel surface via electrostatic attraction, thereby exhibiting good corrosion inhibition performance [15]. As shown in Figure 3b, under the same conditions, when PPA was used alone, its inhibition efficiency was 98.92%, and its corrosion rate was 9.13 g/(m^2^·h). The alcoholic hydroxyl group of PPA remains neutral in strong acid, and the molecule contains no basic nitrogen atom; therefore, PPA exists mainly as a neutral molecule in 20% HCl. Its adsorption relies primarily on the π-electron interactions of the phenyl ring and the alkynyl group, as well as the coordination of the hydroxyl oxygen [11,12]. The two adsorption mechanisms are complementary and can produce a synergistic inhibition effect. To further enhance the inhibition performance, ABQ was compounded with PPA. As displayed in Figure 3c,d, with 0.5% ABQ-PPA, the inhibition performance was significantly improved. At 60 °C and 90 °C, the inhibition efficiencies of ABQ-PPA reached as high as 99.80% and 99.89%, with corrosion rates as low as 1.64 g/(m^2^·h) and 0.97 g/(m^2^·h), respectively, which are well below the maximum allowable corrosion rate stipulated by the SY/T 5405-2019 specification. The significant enhancement in performance after compounding is likely attributable to the fact that the small-molecule PPA can effectively fill the defects and voids in the adsorbed film formed by the main inhibitor, forming a co-adsorbed layer with ABQ molecules on the steel surface and increasing the interfacial coverage density and barrier compactness [16]. Based on the results displayed, the optimal mass ratio of ABQ-PPA was determined to be 4.5:0.5. The effect of the total ABQ-PPA dosage on the inhibition performance for J55 steel was further investigated. As shown in Figure 3e, the inhibition efficiency increased continuously with increasing total dosage; however, the improvement became gradual once the total dosage reached 0.4%. A concentration range of 0.1–0.5 wt% was selected for the subsequent experiments. Taking into account both the inhibition performance and economic considerations, a total concentration of 0.5% was ultimately chosen as the optimized dosage.

### 2.3. Electrochemical Evaluations

#### 2.3.1. Potentiodynamic Polarization Measurements

**Figure 4:**
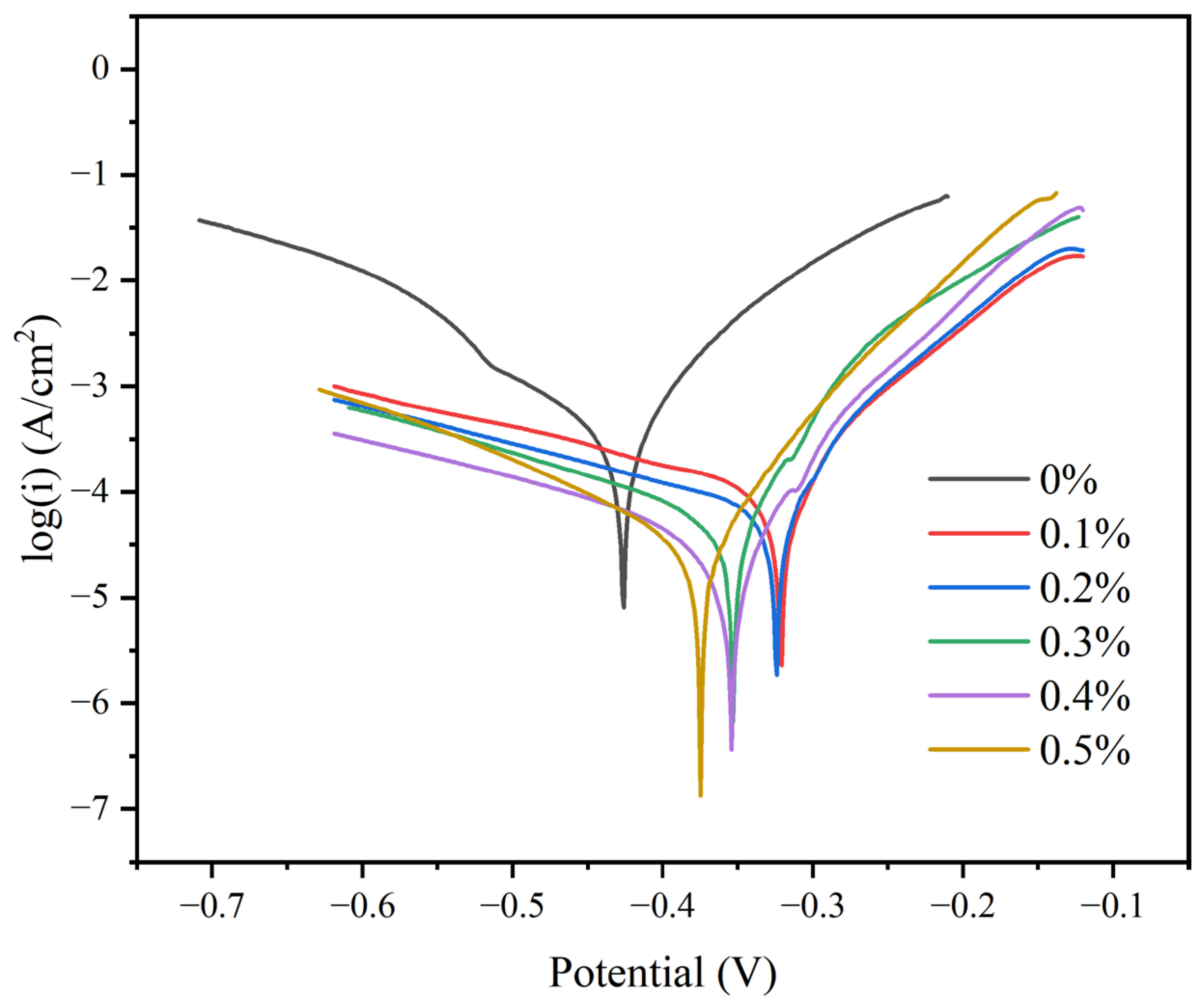
The effect of ABQ-PPA’s dosage on the potentiodynamic polarization for J55 steel in 20% HCl at 25 °C.

The open-circuit potential (OCP) was monitored for 2100 s before each test, and the system was considered to have reached a steady state when the potential fluctuation was less than ±1 mV within the last 400 s. All polarization tests were performed after the OCP had stabilized. Figure 4 presents the potentiodynamic polarization curves of ABQ-PPA for J55 steel in a 20% HCl bulk phase at 25 °C with different dosages. As shown in Figure 4, with increasing total dosage of ABQ-PPA, the corrosion current density (*i*_corr_) exhibited a gradual decreasing trend, which correlated well with the weight-loss results mentioned above. Table 1 summarizes the electrochemical parameters extracted from Tafel extrapolation. As presented in Table 1, compared with the blank solution, an obvious decrease in the corrosion current density (from 4.697 mA/cm^2^ to 0.2698 mA/cm^2^) upon the addition of 0.5 wt% ABQ-PPA was observed, with an inhibition efficiency of 94.26%, fully demonstrating that ABQ-PPA exerted a remarkable inhibitory effect on J55 steel against HCl. Meanwhile, the corrosion potential (*E*_corr_) shifted positively from −405 mV to −354 mV, while the anodic Tafel slope (*β*_a_) decreased from 90 mV·dec^−1^ to 70 mV·dec^−1^, with a positive shift of 51 mV. When a shift of less than 85 mV in corrosion potential before and after the addition of the inhibitor occurs, the inhibitor belongs to mixed-type inhibitors; if a shift exceeding 85 mV occurs, it tends to be identified as an anodic-type or cathodic-type inhibitor [17]. In this study, the positive shift of *E*_corr_ (54 mV) was less than 85 mV, and both *β*_c_ and *β*_a_ exhibited changes, indicating that ABQ-PPA simultaneously inhibits both the anodic and cathodic reactions and can be classified as a mixed-type corrosion inhibitor.

#### 2.3.2. Electrochemical Impedance Spectroscopy (EIS) Evaluation

**Figure 5:**
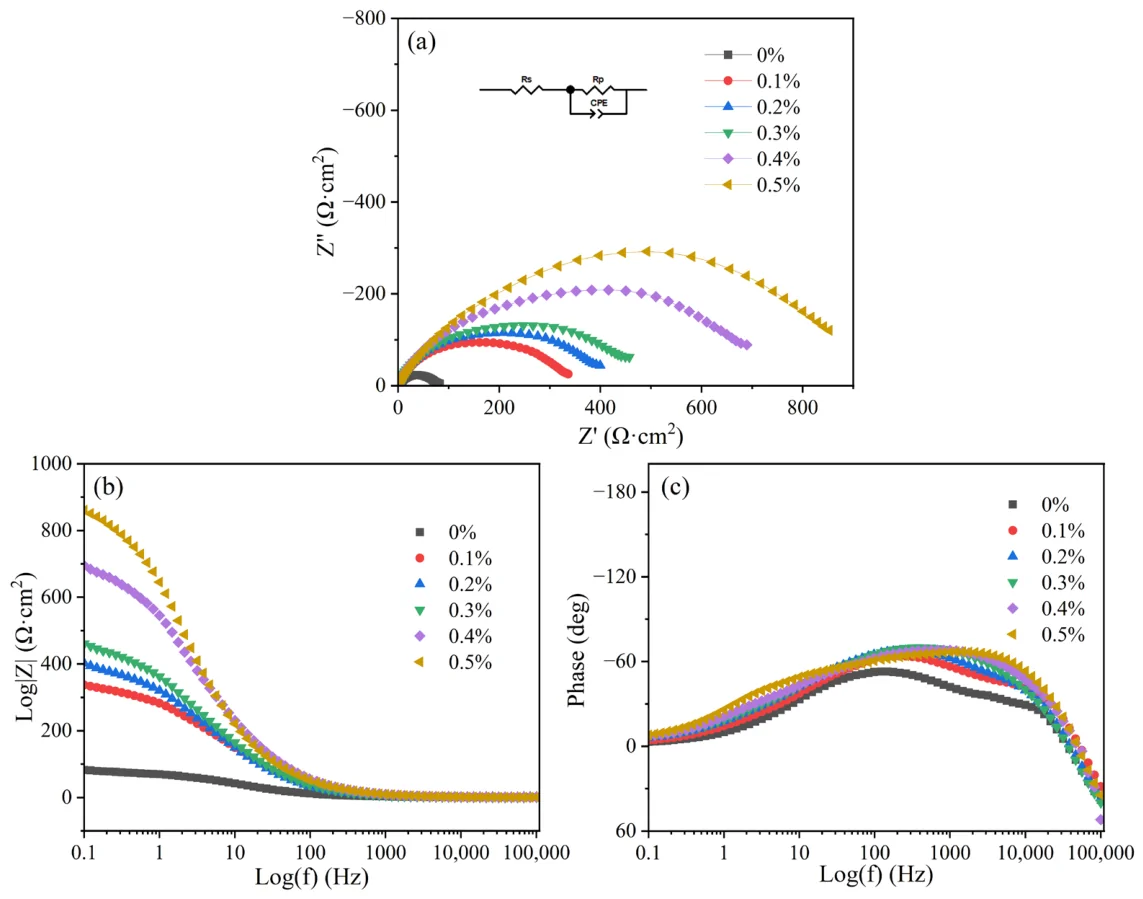
EIS of J55 steel in 20% HCl at 25 °C with various ABQ-PPA dosages: Nyquist plots (inset: equivalent circuit) (**a**), Bode magnitude plots (**b**), and Bode phase angle plots (**c**).

The open-circuit potential (OCP) was monitored for 2100 s before each test, and the system was considered to have reached a steady state when the potential fluctuation was less than ±1 mV within the last 400 s. All EIS measurements were performed after the OCP had stabilized. Figure 5 presents the EIS results for J55 steel protected by ABQ-PPA in 20% HCl at 25 °C. As shown in the Nyquist plots in Figure 5a, the radius of the capacitive loop gradually increased with increasing ABQ-PPA dosage, indicating that the charge transfer resistance (*R*_p_) at the electrode surface was enhanced, thereby effectively suppressing the corrosion reaction [18]. The Bode modulus and phase angle plots, individually displayed in Figure 5b,c, also exhibited consistent trends: with increasing inhibitor dosage, the impedance modulus at low frequencies continuously increased, and the phase angle peaks gradually became larger and shifted toward the lower frequency region. The above observations further confirmed the formation of an ABQ-PPA protective film on the electrode surface, thereby effectively suppressing the corrosion process [19].

Table 2 lists the electrochemical impedance parameters obtained by fitting the EIS data with the equivalent circuit. As shown in Table 2, there was an obvious increase in the charge transfer resistance (*R*_p_) (from 74.75 Ω·cm^2^ without inhibitor to 569.1 Ω·cm^2^ with ABQ-PPA), indicating that the interfacial charge transfer process was markedly hindered. Meanwhile, a decrease in the double-layer capacitance (*C*_dl_) occurred from 1.068 mF·cm^−2^ × 10^−3^ to 0.06437 mF·cm^−2^ × 10^−3^, which may have resulted from the adsorption of inhibitor molecules onto the electrode surface, replacing the originally adsorbed water molecules and decreasing the local dielectric constant at the interface [20]. Meanwhile, the CPE parameter Q decreased from 0.927 × 10^−6^ Ω^−1^·cm^−2^·s^n^ to 0.096 × 10^−6^ Ω^−1^·cm^−2^·s^n^, whereas the CPE exponent n ranged from 0.69 to 0.85. The inhibition efficiency calculated from the impedance parameters reached a maximum of 86.87%, which agreed with the results obtained from the polarization plots and weight-loss tests, further confirming that ABQ-PPA exhibits excellent corrosion inhibition properties for J55 steel in a hydrochloric acid environment.

### 2.4. SEM and EDS Analyses

**Figure 6:**
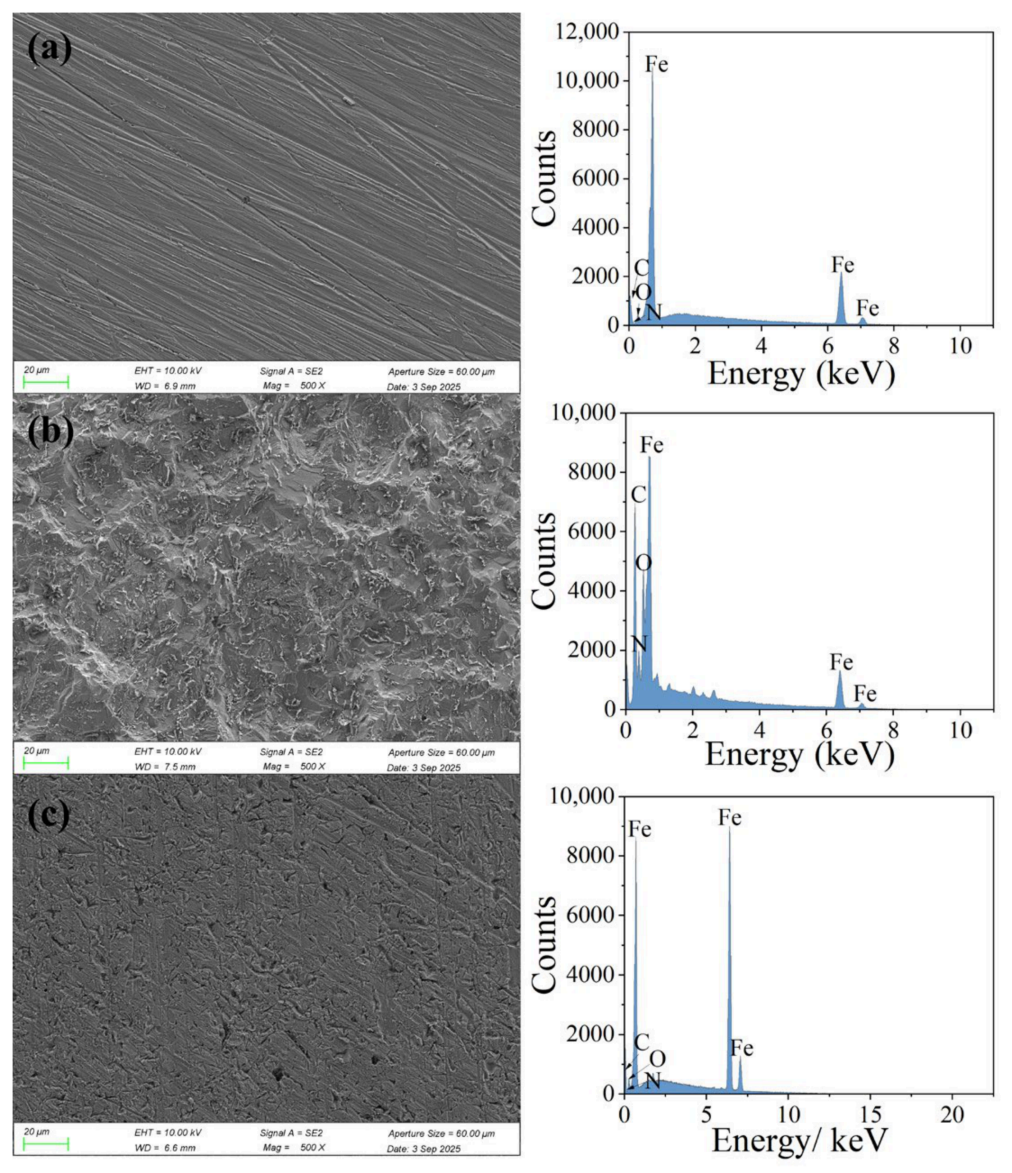
SEM and EDS results of J55 steel specimens under different conditions: surface morphology of pre-polished specimen (**a**), corrosion morphology in 20% HCl at 60 °C (uninhibited) (**b**), surface morphology in 20% HCl at 90 °C with 0.5% ABQ-PPA (**c**).

The SEM and EDS results for J55 steel specimens under different conditions are presented in Figure 6: (a) surface morphology of the pre-polished specimen, (b) corrosion morphology in 20% HCl at 60 °C without an inhibitor, and (c) surface morphology in 20% HCl at 90 °C with 0.5% ABQ-PPA. As shown in Figure 6a, well-defined uniform grinding scratches were observed on the surface of the pre-polished J55 steel specimen, with no corrosion features detected. In Figure 6b, severe corrosion morphology was observed on the steel surface without inhibitor protection, where the scratches were almost completely obliterated, indicating that an intensive corrosive attack was inflicted on the J55 steel substrate by the hydrochloric acid medium. In contrast, in Figure 6c, the introduction of 0.5% ABQ-PPA significantly alleviated surface corrosion, with only minor corrosion traces being visible, while the grinding scratches from the pretreatment stage remained discernible. These observations clearly demonstrated that the inhibitor provided effective protection to J55 steel in the high-temperature HCl environment at 90 °C, effectively hindering corrosion attack from the aggressive medium.

The elemental compositions of the J55 steel surfaces determined by EDS analysis are summarized in Table 3. With ABQ-PPA, the Fe content was found to increase markedly, whereas the O content decreased significantly compared with that under inhibitor-free conditions. The increase in Fe content was ascribed to the suppressed corrosion, which preserved a larger fraction of the substrate surface; the notable reduction in O content suggested that surface corrosion products were substantially diminished. These findings further corroborated that the inhibitor effectively blocked direct contact between the J55 steel surface and H^+^ ions via surface adsorption.

### 2.5. Adsorption Isotherm Analysis

**Figure 7:**
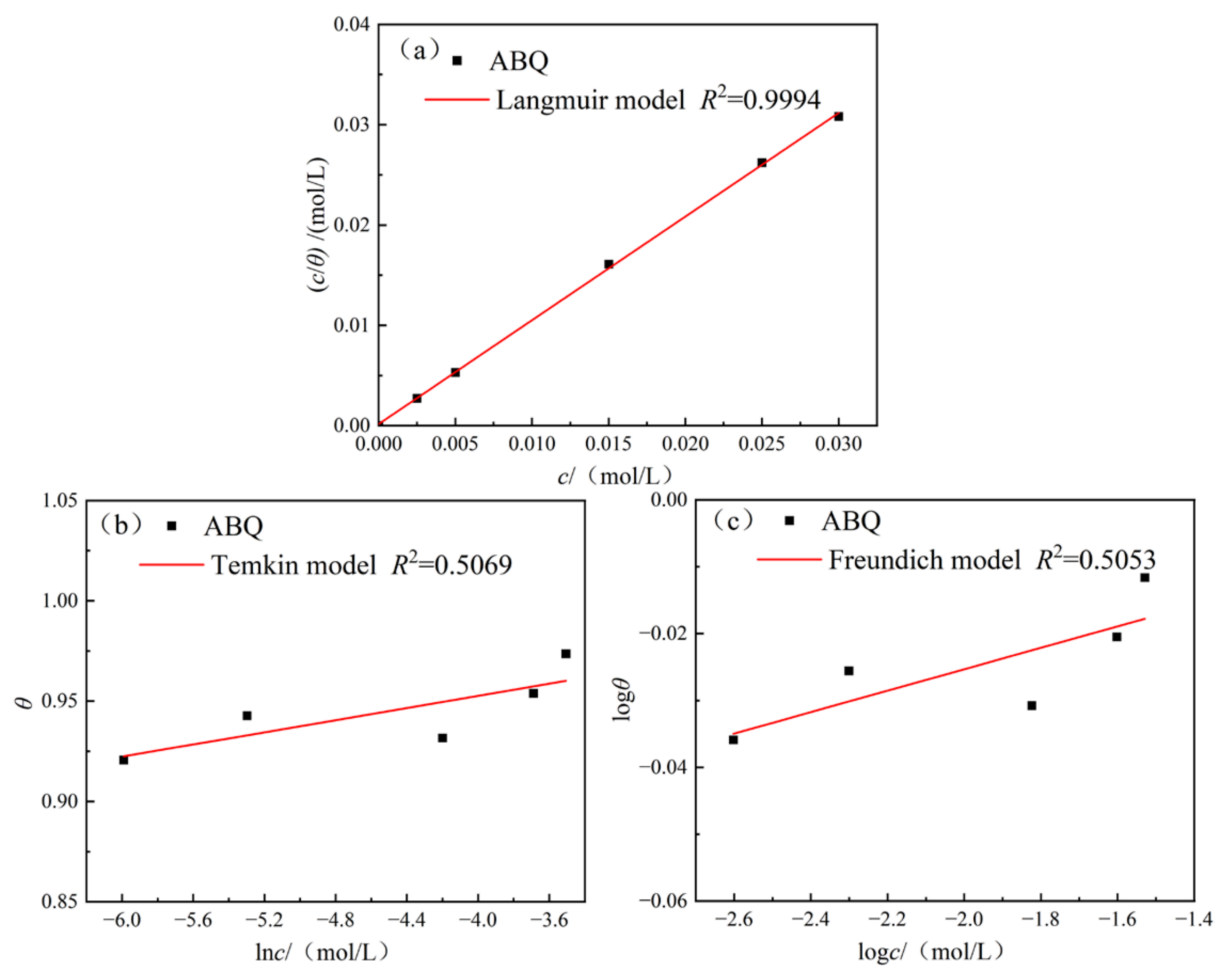
ABQ’s Langmuir (**a**), Temkin (**b**), and Freundlich (**c**) adsorption models on the J55 steel surface.

The adsorption behavior of ABQ on the metal surface is crucial for elucidating its inhibition mechanism and can be quantitatively analyzed using adsorption isotherm models. Based on the inhibition efficiencies obtained from weight-loss measurements for ABQ alone at 60 °C. Figure 7 presents the fitting results of different adsorption isotherm models. Figure 7a, Figure 7b, and Figure 7c correspond to the Langmuir, Temkin, and Freundlich models, respectively. The results showed that the *R*^2^ values of the Langmuir, Temkin, and Freundlich models were 0.9994 (Figure 7a), 0.5069 (Figure 7b), and 0.5053 (Figure 7c), respectively, indicating that the Langmuir model provided the best fit among the tested models and that the adsorption behavior of ABQ could be empirically described by the Langmuir adsorption isotherm model at the macroscopic level. The Langmuir adsorption isotherm equation is expressed as Equation (1):

(1) $$\frac{c}{\theta }=\frac{1}{K_{\mathrm{ads}}}+c$$

where *c* is ABQ’s concentration (mol/L), *θ* is J55 steel’s surface coverage, and *K*_ads_ is ABQ’s adsorption equilibrium constant (L/mol).

The ABQ adsorption isotherm on the J55 steel surface was derived from the linear plots of *c*/*θ* versus c displayed in Figure 7. A satisfactory linear relationship between *c*/*θ* and c was observed, with the regression coefficient (*R*^2^) approaching unity, indicating that the adsorption behavior of ABQ on the J55 steel surface was well fitted by the Langmuir adsorption isotherm in an apparent sense. This finding suggests that the adsorption of ABQ on the J55 steel surface can be approximated as predominantly monolayer adsorption on macroscopically equivalent sites, with lateral interactions being negligible in an averaged sense [21].

The standard adsorption free energy (Δ*G*^0^_ads_) was computed using Equation (2):

(2) $$\Delta G_{\mathrm{ads}}^{0}=-\mathrm{RTln}(C^{*}\cdot K_{\mathrm{ads}})$$

where C^*^ is the adsorption equilibrium constant (L/mol), *R*’s value is 8.314 J/(mol·K), and *T* is the absolute temperature during each measurement (K). Water’s molar concentration was considered to be 55.5 mol/L.

ABQ’s standard adsorption free energy (Δ*G*^0^_ads_) on the J55 steel surface was finally calculated to be −34.83 kJ/mol, which suggested ABQ’s spontaneous adsorption nature. According to the generally accepted criterion, |Δ*G*^0^_ads_| < 20 kJ/mol corresponds to a physisorption mechanism, |Δ*G*^0^_ads_| > 40 kJ/mol corresponds to a chemisorption mechanism, and an intermediate value (20–40 kJ/mol) indicates an adsorption mechanism containing both physisorption and chemisorption processes [22]. In this study, Δ*G*^0^_ads_ was computed to be −34.83 kJ/mol; its absolute value falls within 20–40 kJ/mol, suggesting that the adsorption behavior of ABQ involved a mixed mechanism in which physisorption and chemisorption coexist, which may be caused by the generation of coordination bonds between the ABQ molecules and surface Fe atoms via charge transfer, thereby reinforcing the adsorption affinity between ABQ and the J55 steel surface [23].

### 2.6. MD Simulations

**Figure 8:**
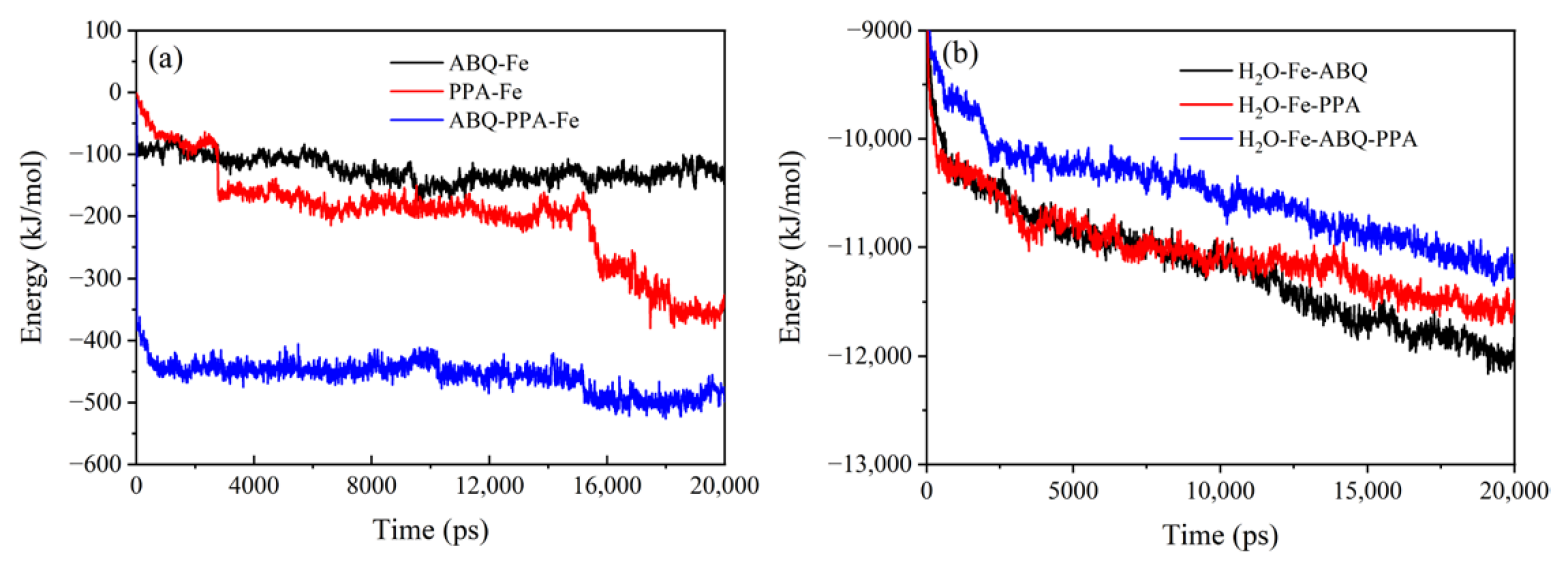
Molecular interaction energies of ABQ, PPA, and water with the Fe substrate versus simulation time in different systems: (**a**) inhibitor–substrate interaction energies; (**b**) water–substrate interaction energies.

Figure 8 illustrates the time evolution of molecular interaction energies between ABQ, PPA, water molecules, and the Fe substrate in various simulation systems. Specifically, Figure 8a presents the interaction energies of the Fe substrate with pure ABQ, pure PPA, and the ABQ-PPA composite system, while Figure 8b displays the interaction energies between water molecules and the Fe substrate in each simulation. As can be seen in Figure 8a, the interaction energies for all three systems (pure ABQ, pure PPA, and the ABQ-PPA mixture) decreased gradually at the initial stage and subsequently leveled off with minor fluctuations throughout the simulation, indicating that adsorption equilibrium was attained in the later stage for all systems. The total interaction energies of pure ABQ, pure PPA, and the ABQ-PPA mixture with the Fe substrate were determined to be −124.894, −424.672, and −491.562 kJ/mol, respectively. After normalization of the interaction energies, the pure ABQ system is expressed per ABQ molecule as −8.921 kJ/mol, the pure PPA system is expressed per PPA molecule as −10.617 kJ/mol, and the mixed system is expressed as a mixture average per molecule as −30.722 kJ/mol. A more negative interaction energy indicates stronger interfacial adsorption attraction. Among these, the ABQ-PPA mixed system possessed the highest absolute interaction energy, considerably surpassing those of the individual systems, which implies a possible synergistic adsorption effect between ABQ and PPA. The co-adsorption of these two molecules contributed to the generation of a more stable protective film, thereby improving both the surface coverage and the interfacial adhesion strength on the Fe substrate [24]. As displayed in Figure 8b, the interaction energies between water molecules and the Fe substrate in all systems also exhibited a decreasing trend followed by stabilization over time. The average water–substrate interaction energies in the pure ABQ, pure PPA, and ABQ-PPA systems were −12,003.3, −11,567.4, and −11,186 kJ/mol, respectively. After normalization of the interaction energies, the average interaction energies per water molecule for the pure ABQ, pure PPA, and mixed systems were calculated to be −2.159, −2.080, and −2.011 kJ/mol, respectively. Notably, the ABQ-PPA mixed system yielded the lowest absolute water–substrate interaction energy, suggesting that the co-adsorbed inhibitor layer most effectively weakened the affinity of water to the steel surface. Furthermore, it is important to clarify the correlation between the MD interaction energies and the experimentally estimated standard adsorption free energy (Δ*G*^0^_ads_). It should be noted that the experimental Δ*G*^0^_ads_ (−34.83 kJ/mol) was derived specifically for pure ABQ. Although the MD potential energy and macroscopic free energy have distinct physical definitions and cannot be directly compared numerically, a qualitative mechanistic correlation exists: the strong negative interaction energy of pure ABQ (−8.921 kJ/mol per molecule) reveals its microscopic driving force for adsorption, which fundamentally explains the spontaneous macroscopic adsorption of ABQ (negative Δ*G*^0^_ads_). More importantly, the normalized per-molecule energies show that the ABQ-PPA mixture (−30.722 kJ/mol) has a significantly stronger binding affinity than pure ABQ and pure PPA. This mechanistic trend strongly supports the synergistic effect, which perfectly aligns with the experimental observation of higher inhibition efficiency.

**Figure 9:**
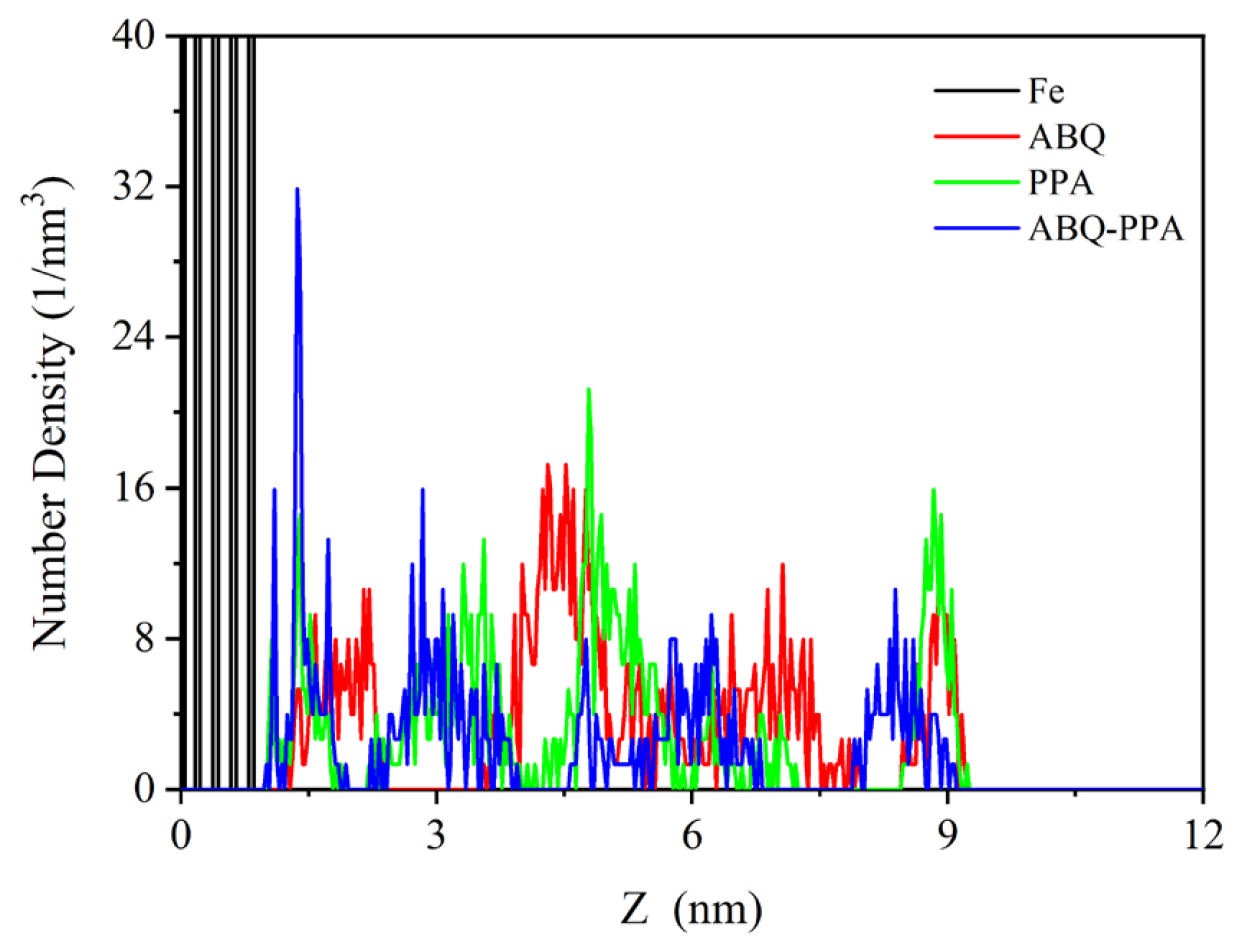
Axial number density distributions of individual molecules in the pure ABQ, pure PPA, and ABQ-PPA mixed molecular simulation systems.

Figure 9 presents the axial number density distribution profiles of various components on the Fe substrate surface in different simulation systems. As shown in Figure 9, in the pure ABQ, pure PPA, and ABQ-PPA mixed systems, the adsorbed molecules all exhibited an aggregation behavior on the Fe substrate surface, and their axial number density profiles displayed obvious characteristic peaks near the Fe surface, indicating that the inhibitor molecules in each system were able to form adsorption films on the Fe surface. A further comparison of the axial number density distributions between the mixed system and the single-component systems revealed that the overall molecular axial number density peak in the ABQ-PPA mixed system was slightly higher than those of the corresponding molecules in the pure ABQ and pure PPA systems, suggesting that ABQ and PPA, after compounding, possessed a stronger aggregation tendency and higher interfacial enrichment on the Fe substrate surface. This finding is in line with the interaction energy calculations shown in Figure 8a, where the ABQ-PPA mixed system exhibited the largest absolute value of interaction energy. Together, these findings confirmed the synergistic adsorption effect between ABQ and PPA, indicating that their combined use promoted an ordered arrangement and compact coverage on the Fe surface, thereby facilitating its protective performance on the substrate surface.

**Figure 10:**
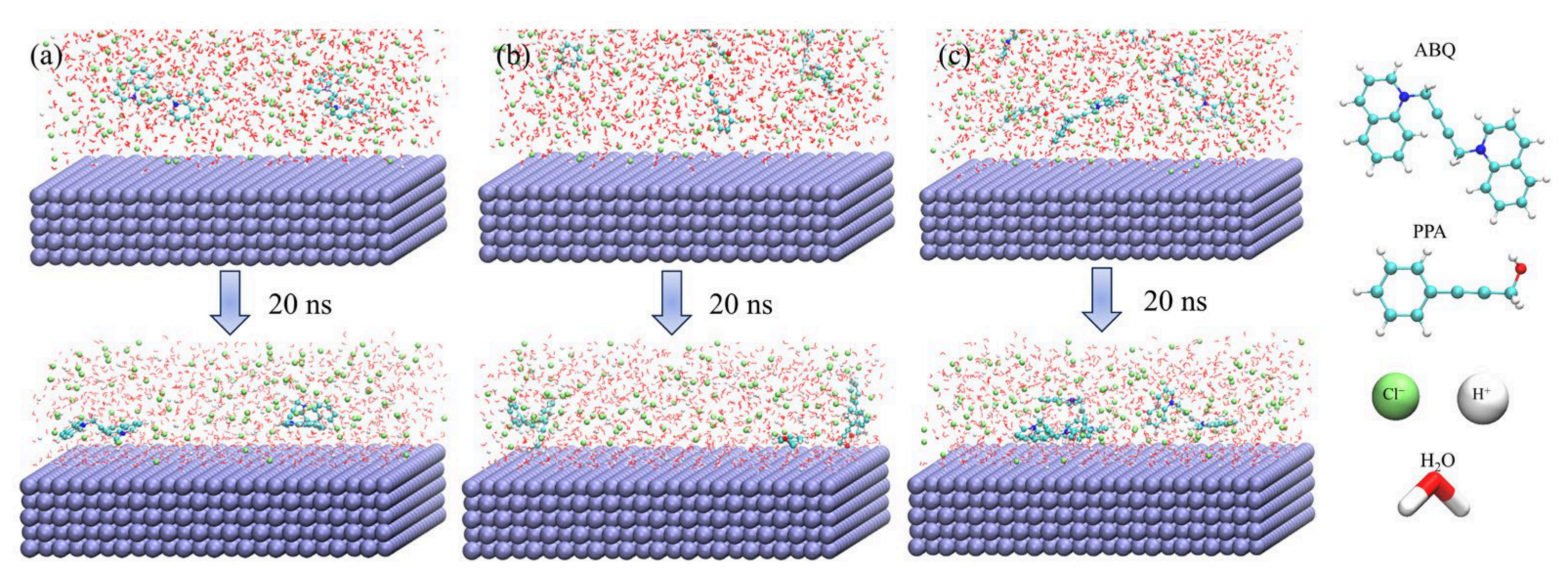
Snapshots of ABQ (**a**), PPA (**b**), and ABQ-PPA (**c**) equilibrium configurations on the Fe substrate surface.

The initial and final equilibrium configurations of each simulation system, visualized using VMD [25] (Visual Molecular Dynamics), are presented in Figure 10. In Figure 10a, the ABQ molecules in the pure ABQ system were initially randomly dispersed in the simulation box. As the simulation progressed, a portion of the molecules gradually migrated toward the Fe substrate and underwent partial aggregation; however, a noticeable spatial gap remained between the molecules and the substrate surface, and no closely packed adsorption layer was formed, indicating that the driving force for ABQ’s migration onto the Fe surface was relatively weak. In the pure PPA system (Figure 10b), the PPA molecules also evolved from an initially dispersed state to a gradually aggregated configuration near the Fe surface. Compared with the pure ABQ system, the PPA system exhibited slightly stronger aggregation and a reduced molecular–substrate distance; however, the adsorbed layer still lacked long-range order and dense surface coverage, implying that the adsorption capacity of PPA alone was insufficient. In striking contrast, as shown in Figure 10c, the ABQ-PPA mixed system displayed a distinctly different behavior: ABQ and PPA molecules collectively migrated toward the Fe surface and underwent pronounced synergistic aggregation at the interface. The two types of molecules approached each other and became interleaved, ultimately forming a structurally compact and densely packed mixed adsorption layer on the Fe surface. The molecular–substrate distance in this mixed system was considerably smaller than that observed in the single-component systems. These results collectively demonstrated that ABQ and PPA were intimately adsorbed onto the Fe surface and, through their synergistic interaction, constructed a stable and robust protective film on the substrate.

## 3. Experimental

### 3.1. Materials and Methods

Quinoline (analytical grade, Shanghai Hansi Chemical Co., Ltd., Shanghai, China), 1,4-dichloro-2-butyne, 3-phenyl-2-propyn-1-ol (analytical grade, Shanghai Aladdin Biochemical Technology Co., Ltd., Shanghai, China), and 37% HCl (Qingdao Xinxier Chemical Co., Ltd., Qingdao, China) were used.

HI650E electrochemical workstation (Shanghai Chenhua Instrument Co., Ltd., Shanghai, China), a Sigma 360 field-emission scanning electron microscope (Carl Zeiss AG, Oberkochen, Germany), and a Nicolet iS50 Fourier transform infrared spectrometer (Thermo Fisher Scientific, Waltham, MA, USA) were used.

### 3.2. Weight-Loss Method

Weight-loss experiments are commonly used to simulate high-temperature corrosion environments encountered in practical applications such as industrial acid pickling. Static weight-loss tests were conducted using J55 steel coupons. The main chemical composition of J55 steel (wt%) is as follows: C, 0.3552; Si, 0.2800; Mn, 1.3080; P, 0.0200; S, 0.0250; Ni, 0.1050; Cu, 0.1560; and Fe, the balance. The surface of each J55 steel coupon was first ground to a smooth finish, and then the coupons were sequentially immersed in petroleum ether and anhydrous ethanol for 5 min each to remove grease and impurities. In the next step, the coupons were weighed, and each coupon’s surface area was calculated. The treated J55 steel coupons were then completely immersed in the corrosion solutions (containing 20% HCl and various concentrations of the corrosion inhibitor) and kept undisturbed at 60 °C (or 90 °C) for 4 h. After the corrosion tests, each coupon was recovered, cleaned, dried, weighed and recorded. Equations (3) and (4) present the calculation methods for corrosion rate and inhibition efficiency, respectively.

(3) $$\nu =\frac{10^{6}(m_{0}-m_{1})}{A\cdot t}$$

(4) $$\eta =\frac{\Delta m_{0}-\Delta m_{1}}{\Delta m_{0}}$$

where *m*_0_ and *m*_1_ are the masses of the steel coupon before and after weight loss, respectively (g); *A* is the surface area of each J55 steel coupon (cm^2^); *t* is the corrosion time (h); Δ*m*_0_ is the mass loss of each steel coupon without the inhibitor’s protection (g); and Δ*m*_1_ is the mass loss of the steel coupon with the inhibitor’s protection (g).

### 3.3. Electrochemical Measurements

Electrochemical evaluations were carried out on a CHI650E electrochemical workstation mounted with a standard three-electrode system and were performed at 25 °C. At this temperature, the thermodynamic and kinetic states of the system are stable, and the solution composition and electrode surface state can be readily maintained constant during the measurement, thereby enabling reliable steady-state electrochemical parameters to be obtained. The testing system consisted of a J55 steel working electrode (1 cm × 1 cm), a platinum sheet auxiliary electrode, and a saturated calomel reference electrode (SCE). Electrochemical impedance spectroscopy (EIS) was conducted at the open-circuit potential at 100 kHz–0.1 Hz with an AC perturbation signal amplitude of 5 mV. Potentiodynamic polarization measurements were conducted at ±250 mV relative to the open-circuit potential, during which the scan rate was kept at 1 mV/s. The inhibition efficiencies were calculated from Equations (5) and (6), respectively.

(5) $$\begin{array}{l} \eta_{T}=\frac{i^{0}-i}{i^{0}}\times 100\% \end{array}$$

(6) $$\eta_{E}=\frac{R_{p}-R_{p}^{0}}{R_{p}^{0}}\times 100\%$$

where *i*^0^ and *i* are the corrosion current densities under the corresponding conditions (A/cm^2^), and *R*^0^_p_ and *R*_p_ are the charge transfer resistances without and with the inhibitor’s protection, respectively (Ω·cm^2^).

### 3.4. Surface Morphology Observation

To observe changes in surface morphology and composition before and after weight loss, three J55 steel specimens were observed and characterized by scanning electron microscopy (SEM-EDS), including the pretreated blank specimen, the specimen corroded at 60 °C without an inhibitor, and the specimen corroded at 90 °C with the protection of 0.5% ABQ-PPA inhibitor.

### 3.5. Molecular Dynamics (MD) Simulation

Molecular dynamics (MD) simulations were performed at 363.15 K (90 °C), which corresponds to the highest temperature used in the weight-loss experiments, aiming to reveal the adsorption behavior and interaction mechanism of the inhibitor on the Fe surface at the molecular level. The calculation of ABQ’s and PPA’s atomic charges of molecules was carried out with the Gaussian 16 program at the B3LYP/6-311g(d,p) level of theory [26]. The bond, angle, and dihedral parameters for each molecule were comprehensively assigned based on the OPLS-AA all-atom force field in conjunction with the AuToFF tool, while the force field parameters from the UFF force field were adopted for the Fe(110) surface. The Fe(110) slab had dimensions of 4.86444 × 5.15952 × 0.81074 nm and consisted of 5 layers with a total of 2160 Fe atoms; its surface area was 25.09817 nm^2^. It was placed in a 4.86444 × 5.15952 × 15 nm simulation box with a vacuum layer thickness of 5 nm. Periodic boundary conditions were applied in all three directions (x, y, and z). The aqueous phase contained 5560 H_2_O molecules and 684 HCl molecules (20% HCl). Based on the optimized formulation (total concentration of 0.5%, consisting of 0.45% ABQ + 0.05% PPA), three systems were constructed: 14 ABQ molecules, 40 PPA molecules, and 12 ABQ + 4 PPA molecules. All MD simulations were run using Gromacs 2021. Prior to the simulations, energy minimization was conducted using the steepest descent method. The force convergence threshold was 1 kJ·mol^−1^·nm^−1^, and the maximum step size was 0.002 ps. The leap-frog algorithm was utilized for the integration of each motion equation. The simulations were run under the NVT ensemble for 20 ns, during which the Fe(110) substrate was kept frozen to maintain structural stability. Each run was kept at 363.15 K using the V-rescale thermostat, while the cutoff radius was 1.5 nm.

## 4. Conclusions

In this study, ABQ, a corrosion inhibitor molecule containing both a quinoline ring and alkynyl groups, exhibited good solubility in 20% HCl. The corrosion inhibition performance and adsorption mechanism of ABQ were systematically investigated. An optimized ABQ-PPA complex (total concentration: 0.5%, 0.45% ABQ + 0.05% PPA; mass ratio: 4.5: 0.5) was developed for J55 steel in a hydrochloric acid medium:

(1) The ABQ-PPA composite inhibitor exhibited excellent protection performance for J55 steel against 20% HCl, yielding an inhibition efficiency of up to 99.89%. The 0.5% ABQ-PPA simultaneously suppressed both anodic and cathodic reaction processes, behaving as a mixed-type inhibitor with predominant anodic inhibition.
(2) Adsorption isotherm analysis revealed the Langmuir adsorption behavior of ABQ on the J55 steel surface, involving a mixed adsorption mode with both physisorption and chemisorption.
(3) MD simulations revealed that ABQ and PPA formed a compact and ordered mixed adsorption layer between the solution phase and the Fe surface through synergistic effects, which effectively displaced interfacial water molecules and blocked the attack of corrosive species.

## Figures and Tables

**Table 1:** Polarization curve fitting data for J55 steel protected by ABQ-PPA in 20% HCl at 25 °C.

ABQ-PPA (wt%)	*E*_corr_ (mV)	*i*_corr_ (mA·cm^−2^)	*−β*_c_ (mV·dec^−1^)	*β*_a_ (mV·dec^−1^)	*η*_T_ (%)
0.0	−405	4.697	137	90	/
0.1	−321	1.496	280	88	68.15
0.2	−324	1.251	269	84	73.37
0.3	−353	0.7898	228	71	83.19
0.4	−375	0.3889	152	64	91.72
0.5	−354	0.2698	215	70	94.26

**Table 2:** EIS fitting parameters of J55 steel in 20% HCl at 25 °C with various ABQ-PPA dosages.

ABQ-PPA (wt%)	*R*_s_ (Ω·cm^2^)	*R*_p_ (Ω·cm^2^)	CPE		*C*_dl_ (mF·cm^−2^ × 10^−3^)	*χ* ^2^	*η*_E_ (%)
			*Q* (Ω^−1^·cm^−2^·s^n^ × 10^−6^)	n			
0.0	0.975	74.75	0.927	0.69	1.068	0.00059	/
0.1	0.861	277.2	0.182	0.82	0.095	0.00071	73.03
0.2	0.812	287	0.174	0.76	0.086	0.00052	73.95
0.3	0.809	294.3	0.118	0.85	0.077	0.00087	74.60
0.4	0.880	450.9	0.125	0.79	0.076	0.00073	83.42
0.5	0.717	569.1	0.096	0.82	0.064	0.00079	86.87

**Table 3:** EDS elemental composition of J55 steel surfaces.

J55 Steel Coupon	W, %			
	C	N	O	Fe
Pretreated	2.57	0.01	0.35	97.07
Without inhibitor	24.35	6.02	6.41	63.22
With 0.5% ABQ-PPA at 90 °C	9.25	0.01	1.51	89.23

## Data Availability

The raw data supporting the conclusions of this article will be made available by the authors on request.
